# Raising complex public health challenges on local government agendas: a Norwegian case study

**DOI:** 10.1186/s12961-025-01347-3

**Published:** 2025-06-18

**Authors:** Kristine Løkås Vigsnes, Charlotte Kiland, Harry Rutter, Eirik Abildsnes

**Affiliations:** 1 Department of Nutrition and Public Health, Faculty of Health and Sport Science, University of Agder, Kristiansand, Norway; 2 Department of Research and Innovation, Kristiansand Municipality, Kristiansand, Norway; 3 Department of Political Science and Management, Faculty of Social Sciences, University of Agder, Kristiansand, Norway; 4 Department of Social and Policy Sciences, University of Bath, Bath, United Kingdom; 5 Institute of Health and Society, Department of Community Medicine and Global Health, University of Oslo, Oslo, Norway

**Keywords:** Agenda setting, Public health, Policy, Complexity, Policy entrepreneurs, Low politics

## Abstract

**Background:**

Public health challenges, ranging from noncommunicable disease prevention to pandemic preparedness, involve both policymaking and the handling of complexity at local, national or global level. The complexity of public health challenges arises from uncertain knowledge, hidden mechanisms, differing conceptual models and multiple stakeholders, making it difficult to identify and agree on problem definitions and potential solutions. This case study aims to provide insights into how complex public health challenges are addressed within local government agendas. We use theories of policy entrepreneurs and agenda setting to explore the processes behind the agenda setting of a public health programme for work inclusion.

**Methods:**

We conducted an in-depth case study in a local government context. Data from interviews with key informants and political documents were triangulated and analysed using deductive thematic content analysis, framed within Kingdon’s agenda-setting theory and theories of policy entrepreneurs.

**Results:**

We identified problem framing, the involvement of multiple actors, and turbulent surroundings as influential factors for agenda setting of complex public health challenges in local government. We further identified the significance of the level of influence of policy entrepreneurs’ positions, their coalitions and maintaining agenda control as core mechanisms for enabling agenda setting. We found that strategic manoeuvres of policy entrepreneurs were crucial to handle and manoeuvre complexity within the multiple streams described by Kingdon.

**Conclusions:**

This article contributes knowledge of how complex public health problems receive attention and how these issues are shaped and defined, placed on governmental agendas, and paired with solutions. The article contributes important insights for understanding policy responses to complex public health problems and how policy entrepreneurs strategically manoeuvre within the streams of policy development to create windows of opportunity and maintain agenda control. This is important knowledge to support agenda setting for public health challenges and to realize policies to improve public health.

**Supplementary Information:**

The online version contains supplementary material available at 10.1186/s12961-025-01347-3.

## Background

People’s health is influenced by various factors, including political, social, economic and physical environments. Consequently, the health of populations is influenced by multisectoral policies [1–3]. Public health policies address complex societal challenges such as health inequality, poverty and poor living conditions. Such public health challenges are often characterized by an uncertain knowledge base and hidden mechanisms, making it difficult to identify solutions and reach common agreement on solutions. As public health challenges are complex, predicting the outcomes of public health policies can be challenging and characterized by uncertainty [4]. As a result, complex public health issues may be neglected or sidelined from policy agendas, making it difficult to prioritize them on the governmental level [1, 2]. In this article we investigate agenda setting of young people who are not in education, employment or training (NEET) and, more specifically, how such a complex public health challenge is organized on the agenda in a local government setting.

Agenda setting in governmental organisations can be defined as the issue-sorting phase in a public policy process [3]. This early stage in the process is critical for generating political priority for an issue and setting the direction for further policy development. In agenda-setting literature, a major argument is that the agenda is significantly impacted by how people make sense of, define, process, pay attention to or neglect policy problems [3, 5, 6]. Multiple actors’ efforts to influence agenda setting and make sense of complexity seem especially relevant regarding public health challenges because such issues are characterised by increased expectations of multisectoral cooperation, including multiple actors with agenda-setting power. 

Agenda setting is closely related to priority setting and discussions on the distribution and allocation of scarce resources and money [3]. However, the agenda setting of complex public health issues has been given limited attention. As a result, we have accumulated little systematic knowledge of how public health issues are raised on governmental agendas. In this article, we build on the work of Béland and Katapally [7] and their critical analysis of literature on policy change in a local government setting. Identifying how public health issues are framed and raised on local governmental agendas and what actors do to alter health policy priorities may lead to better understanding of the mechanisms that shape the effective development of population health policies. Specifically, we empirically investigate agenda setting in relation to a complex public health issue – namely, how local governments support young people who are not in employment, education or training (NEET) [8]. Turnbull and Hoppe [5] argue that complex societal challenges cannot be understood in isolation but must be viewed within the context of policymaking and the decision-makers and practitioners who deal with them. Our aim is to advance the concept of policy entrepreneurs in public health, acknowledging the complexity within which shaping and formulating public health issues takes place. From the agenda-setting literature, we focus on Kingdon’s [6] argument about how policy entrepreneurs navigate policy streams and exploit the so-called window of opportunity to frame and formulate policy issues for political agendas. Further, we investigate how policy entrepreneurs handle agenda setting in a complex setting (i.e. stronger external pressure for collaboration, multiple external stakeholders, increased organisational noise and complex societal challenges). In this context, local governments are expected to closely interact with residents and address their needs through consensus-driven politics.

This paper has both theoretical and empirical goals. First, we address the potential contribution of our work by applying the agenda-setting theory and the complexity of public health challenges to better understand the process of shaping and framing policy issues in public health. Second, we investigate the role of policy entrepreneurs in this process.

We address the following research question: How is agenda setting of complex public health issues handled in local government policymaking?

### Context

The case study took place in a Norwegian municipality, a regional capital with 115 000 citizens. We refer to it by a fictitious name, Greenville. In Norway, municipalities fall under the Public Health Act [9], which defines local governments as responsible for public health issues. Municipalities are local authorities responsible for a wide range of welfare services for their citizens, such as public health, health planning, primary health care and social services, welfare and primary education. Municipalities provide services to people where they live their everyday lives and are important for addressing the social determinants of health [10]. A high level of autonomy enables municipalities not only to organise and implement services but also to develop and test new interventions [11, 12]. Municipalities in the Nordic countries represent complex adaptive systems consisting of multiple interconnected sectors and levels of government. This makes the municipal setting particularly relevant for investigating how complex public health challenges are raised on local governmental agendas.

Norway can be described as a multi-party system with large variation in municipality sizes, ranging from 200 to 700 000 inhabitants. Most of the 356 Norwegian municipalities have the same political structure since the introduction of the aldermanic system [13] in 1837. This implies an organisational form endeavouring proportional political representation in all committees. Political positions are assigned from key political parties according to their strength in the local council. The model has been characterised as a typical so-called consensus democracy characterized by elected politicians who try to reach consensus across party political cleavages as opposed to a so-called majority democracy [14]. City councils are elected for a period of 4 years, and senior elected officials are chosen by city council members for that period. Senior elected officials chair political meetings, have speaking and proposal rights, participate in political debates and express their views on various matters. Still, political leaders, such as senior elected officials, operate within a system that provides few formal powers and decision-making authority. They are expected to discuss and negotiate with others to reach consensus and collectively make decisions. Local politicians are dependent on their reputational power [15], supporters and ability to build so-called soft power within networks [16].

In this study, we examined policymaking in terms of a local work inclusion programme addressing young people who are experiencing NEET. Norway has a low proportion of individuals who are NEET compared with countries in the European Union, but Norwegian young people in NEET situations have similar severe challenges related to health and low education to those elsewhere [8, 11, 17]. The challenges faced by people who experience NEET are situated in a complex ecosystem involving welfare services, education, private and public employers, nongovernmental organizations (NGOs), politicians, leaders and practitioners. This case is highly relevant for investigating how complexity is handled in agenda setting.

### Raising public health challenges on governmental agendas

Goldsmith and Larsen [18] have reviewed local political leadership in the Nordic countries. They describe the local government in the Nordic area as small scale and frequently rural, strongly partisan, relying on a strong tradition of consensual, corporatist style of decision-making. A social democratic consensus places a stress on the continued production and delivery of high-quality welfare state services, characterised by a style of local leadership which is collective in nature and in which the strong mayor concept is rare. Lately, this characterization has been challenged slightly. While the aldermanic model [13] encourages consensus building, Norwegian local politics have increasingly been associated with conflicts and decision-making characterised more by compromises than consensus [19]. Different from the typical party-political conflicts at the national level, conflicts at the local level in Norway tend to relate more to specific local issues and with less references to national government parties or the ideological party-line [20] as well as increased political fragmentation [21]. Despite local variations of increased political dissent and antagonistic behaviour [22], most Norwegian local politicians characterise the political atmosphere more in terms of being collaborative than consensus based [15]. This might also trigger the so-called scope of conflict in agenda setting as it relates to interests and disagreements about which issues that are important should be prioritized and placed on the political agenda (p. 49). Conflict related to the political agenda is interpreted as a major political activity by politicians involved in local politics [15].

The principle of Health in All Policies (HiAP) recognises that health is influenced by factors beyond health, including political, social and economic factors and physical environments [23], and applies to multisectoral policies and action among health and non-health sectors [24]. This approach to population health challenges requires responses from the whole of society. For local governments, this involves critical collaborations across relevant sectors as well as partnerships between stakeholders from civil society, academia and government. By advancing the HiAP principle and acknowledging the structural determinants of health, public health challenges enter the political arena. As Kickbusch [23] describes:

Politics is messy and once a health issue enters the political arena, it becomes part of a larger agenda and the mix of evidence, interests and ideology can produce strange bed fellows and surprising compromises; health can bring political adversaries together or become an instrument to emphasize differences and gain popular support and votes (p. 199).

Agenda setting involves the mobilisation of particular actors involved in the policy process. This could involve many different types of actors, from experts to people associated with interest groups, social movements, the media and political parties. Agents and lobbyists representing commercial industries and interests could become relevant as well. A multisectoral approach to public health inevitably increases the complexity and number of stakeholders involved in agenda setting.

The complexity of public health issues entails increased expectations of collaborative governance and cross-sectoral co-design between local governments and stakeholders outside the municipal organization. The concept of so-called interactive political leadership introduces a way for politicians to involve citizens in addressing complex societal challenges. Interactive leadership is a political strategy where elected politicians may aim for legitimacy through initiating co-creation [25]. By applying interactive leadership, Torfing and Sørensen [25] argue that elected politicians can benefit from initiating, orchestrating and engaging in co-creation processes, as their involving of citizens and stakeholders in a co-creation process may increase legitimacy. This illustrates the importance of increasing our knowledge of how complex public health problems receive attention and how these issues are shaped and defined [26], placed on the agenda [5, 6] and paired with solutions [6].

### NEET as low politics

Interesting contributions in the agenda-setting literature differentiate between high and low politics [27–29]. Concerns related to urgent matters are referred to as high politics [27, 28], such as the economy and medical treatment. Other less urgent issues are typically defined as low politics [27, 29], emphasising health-promoting measures especially of a wide-ranging, societal nature. NEET can be understood as a low politics issue owing to its multifaceted nature as a public health challenge. It involves intricate causal relationships influenced by various factors. Addressing NEET situations requires sustained investments and efforts over time, even though the outcomes are uncertain. In local governments characterised by performance management systems, low politics issues such as NEET seem more insensitive to policy measures than more urgent high politics issues and, therefore, remain low-profile issues on governmental agendas. Policymakers may consider the complexity, uncertainty and time lag between cause and effect to decrease the possible benefits of low politics issues. Eventually, the lack of noticeable benefits or effects within the remaining cabinet period can lead to a lack of interest and willingness among politicians to deal with such issues [27]. The willingness of politicians and seriousness of the problem tend to be underestimated – or, as Willemsen [27] put it, “out of sight is out of mind” (p. 281). Therefore, it is especially challenging to raise low politics issues on a governmental agenda.

### The role of policy entrepreneurs in agenda setting

Raising low politics issues – particularly on local governmental agendas – actualises the importance of policy entrepreneurs. Kingdon [6] was concerned with why certain issues or themes are raised on an agenda while others are neglected. Equally important is the question of why actors, both inside and outside the decision-making apparatus, pay attention to some issues at the expense of others. The multiple-stream model constitutes a useful tool for understanding agenda setting through three partly separate streams: problems, solutions and policies. The problem stream refers to problems in which authorities take an interest. The possibility that an issue will be set on an agenda increases significantly if it is linked to an important problem [6]. If political entrepreneurs manage to connect an issue to a problem (e.g. connecting NEET situations to poor living conditions or inequality in health), it is more likely that the issue will be selected over the others and raised on the agenda. The second stream, the solution stream, represents the ideas and proposed solutions put forward by the actors involved, preferably as a solution to the problem. Political entrepreneurs have an important role in bringing the so-called softening phase to an end, making the general public and actors involved receptive to the new solution. The third stream, the political stream, relates to political conditions and a combination of the national mood, the elected officials involved in the decision-making process and interest groups [6]. People in and around authority perceive a national mood that more concretely reflects the mood of public opinion. In a municipality, a combination of the national and the local mood, pressure groups and an upcoming local election can force an issue onto the agenda, thereby making it difficult for the city council to devote its attention to other policy issues. The streams exist in parallel, but at a critical point, they can be linked by a policy entrepreneur.

Kingdon [6] defined policy entrepreneurs as actors who work strategically to set agendas by investing resources and effort into presenting their ideas to various forums, hoping that their favourite solutions or so-called pet proposals are captured in the political decision-making process. Thus, they keep an eye on what is happening within the various streams [6]. Policy entrepreneurs have an important role in connecting streams. They keep their alternatives for themselves, waiting for the occurrence of a problem that makes it possible to gain traction for a proposal. In other words, the problem and solution need to fit the prevailing political climate. According to Kingdon’s [6] multiple-streams framework, the policy entrepreneur is most important in exploiting a window of opportunity by raising an issue on the formal governmental agenda. A window of opportunity represents a possibility for certain actors to launch their alternatives or solutions or to draw attention to specific problems. It occurs when the three streams are connected and only stays open for a short period of time. Sometimes it is predictable, such as owing to elections, but it can often occur unexpectedly, especially as a result of a crisis. Timing is crucial, and they manage to exploit the opportunity to address their preferred policy solution to the problem while the policy window is open [6].

Policy entrepreneurs are influential political and social actors who are central to determining which issues are moved onto and off policy agendas with new or existing solutions [30]. Classically, policy entrepreneurs are described as agents of policy change who have the necessary knowledge, endurance, power and luck to enable them to grab the right opportunities when they occur [6]. Cairney [30] has described how policy entrepreneurs invest effort and time wisely for future achievement and how policy entrepreneurship is a strategic endeavour for which persistence and nudging may be applied. Policy entrepreneurs draw attention to some problems over others, and they engage by telling simplified stories about complex issues built upon both facts and emotions, specific perspectives or even lies [31]. Lynch [32] describes how policy entrepreneurs frame issues in certain ways to justify public intervention. Béland and Cox [33] showed how policy entrepreneurs can shape policy ideas as so-called coalition magnets, building coalitions around particular policy solutions to pressure government officials to adopt these solutions. In the Norwegian context, poor living conditions are an example of a coalition magnet that unites actors in a common effort, where there has been cross-party consensus at the national level, and policy has been developed across departmental areas through a National Strategy to Reduce Child Poverty (2015–2017), followed by a Strategy for Children and Youth in Low-income Families (2020–2023) [34]. Policy entrepreneurs develop feasible solutions that they introduce to policymakers when a window of opportunity opens, and they are also capable of creating windows of opportunity through their policy networking and participation in the policymaking landscape [30]. Although policy entrepreneurs act strategically [30], policy change is not influenced only by human agency, since the complex nature of institutions influences their actions and ability to act. Institutional features within an organisation may constrain a policy entrepreneur – in our case, it is the municipality through institutional barriers, also called embedded agency [35]. 

## Methods

### Study design

We applied an in-depth, single-case design [36] to empirically investigate agenda setting of NEET as a public health challenge in a local government context. The Work Inclusion Programme was identified as a relevant case for our research questions, as the agenda setting of a complex public health challenge took place in the complex context of municipal bureaucracy.

### Data collection

First, we collected documents related to the agenda-setting process (Supplementary File 1: Table 1), including political documents, political presentations and the assignment committee’s report, to achieve broad insight into agenda setting and the formulation and framing of the issue of NEET. Second, to obtain direct insights into actors’ motivations, actions and interactions, we conducted 13 in-depth interviews. The informants were political and administrative leaders considered to be elite and key informants with specific and detailed knowledge about the case [37]. They were identified through non-probability purposive sampling [38], whereby informants were identified through their central roles in the policy development process. The interviewees constituted a strategic set of political and administrative leaders and central actors who participated in the policy development process. All interviews were conducted by K.L.V.

Table 1 presents the informants included in this study. Informants are anonymised and numbered. Their formal positions are described.

**Table 1:** Overview of informants

Informant no.	Role, affiliation
Informant 1	Senior elected official
Informant 2	Senior executive
Informant 3	Senior executive
Informant 4	External stakeholder
Informant 5	Senior executive
Informant 6	External stakeholder
Informant 7	Senior executive
Informant 8	Senior elected official
Informant 9	Senior executive
Informant 10	Senior executive
Informant 11	Senior executive
Informant 12	Senior executive
Informant 13	Senior executive

The interviews were semi-structured to enhance information retrieval; they lasted between 45 min and 120 min and were conducted in person during 2023. They covered the following main topics: roles, involvement, agenda setting, policy formulation, discussions, agreements and disagreements, actors, interactions between politics and administration and process description. All interviews were audio recorded and subsequently transcribed verbatim by the first author.

### Data analysis

The analysing process was conducted as a thematic analysis, inspired by Braun and Clarke [39]. We applied a deductive approach where the thematic analysis was mainly theoretically driven. Data were coded deductively according to predefined theoretical themes of problem stream, solution stream, policy stream, and policy entrepreneurs on the basis of the theoretical framework of agenda-setting theory and Kingdon’s multiple-streams theory. In addition, we performed inductive coding to further explore the empirical findings and constructed the following themes: Assignment committee, Municipality 3.0, Politics, Surrounding Turbulence and Implementation.

The ambition was to investigate how the streams unfold in an agenda-setting process in local government context. Themes and analysed codes are described in Supplementary File 2.

We triangulated data sources [40] and integrated interview data and data from documents in the analysis. NVivo was used in the analysing process and to combine the different types of information [41].

### Ethics

All data were pseudonymised. All informants were informed both verbally and in writing of the risk that specific professional roles could be recognised, and all provided written consent to participation. The project was approved by the Ethics Committee of the Faculty of Health and Sport Sciences at the University of Agder and by the Norwegian Agency for Shared Services in Education and Research (reference no. 248715).

## Results

### The idea of system change

The initiating senior elected official was described as ambitious when he in 2018 proposed a policy change to address young people experiencing NEET situations (informants 5 and 6). The challenge of NEET had been problematised nationally by the Norwegian government [42] and internationally by the Organization for Economic Cooperation and Development (OECD; 2018). The Minister of Education as well as the Director of Health had expressed their concerns about NEET in the media [43, 44], but there was a lack of policies and interventions at the national level. The senior elected official launched the system change initiative during the third year of the 4-year elective period. Addressing NEET as a public health challenge that required system change was strongly inspired by a Danish municipality that had succeeded in addressing NEET as a system challenge and conducted organisational and structural changes to improve work inclusion (informants 1, 2, 5, 6 and 7).

*And we were inspired by what they had done in [the Danish municipality]. They managed to make a power investment, involving widely, and they had clear objectives* (Informant 8, senior elected official).

The Danish idea was identified as a potential solution to Greenville’s challenge. The solution was based on an understanding of NEET– situations were caused by systemic barriers and not as individual problems (informants 1, 2, 4 and 8). The motivation for addressing the challenge was the need for increased workforce in the local business life and more taxpayers in the community (informants 1 and 8). The Danish inspiration was motivated by the so-called Municipality 3.0 or Smart City approach based on the idea of a more integrated way of managing cities and public–private partnerships to handle complex problems [45]. In the budget priority process of December 2018 (Document 1), it was suggested that an assignment committee should be established to investigate how to approach the public health challenge of Greenville’s young people experiencing NEET. The city council unanimously supported the initiative, and the administration was asked to establish an assignment committee on behalf of the city council (Document 2). The Local Government Act [46] gives municipalities in Norway extensive freedom when it comes to involving citizens in local democratic processes. Establishing committees for municipal and county purposes provides the opportunity for the committees to be operational for a short time and to have detailed tasks with the purposes of assessing and offering opinions or advice on specific matters [47]. This opportunity was taken up in this case; an assignment committee was established. Involving multiple actors in a design-thinking process was motivated by an ambition to innovate (informants 1, 2, 6 and 8).

### Involvement of multiple actors

The assignment committee was broadly composed of actors from different positions in the field of work inclusion, the local business community and user representatives. In total, the committee consisted of 28 members (Document 4). All participants were formally invited by the administration after consultation and in agreement with the municipal executive committee (informants 1, 2 and 6). An external stakeholder was engaged as the committee leader, and a consultant agency was hired to facilitate a design-thinking process [48] for the committee. Design thinking is a method used to develop user-centric understanding of challenges and potential scenarios for cities aiming at innovation and smartness [45].

The strategy of establishing an assignment committee involving multiple actors from local business and volunteer organisations was motivated by an understanding of NEET as a complex challenge that could not be dictated solely by political decisions or administrative strategies:

*You sit on the city council and decide what the Norwegian Labour and Welfare Administration should do, what the business community should do or what labour market companies should do. It’s such a naive management model – just as stupid as the city council deciding that we will now establish a number of companies in [Greenville]. A business strategy. Completely excessive faith in one’s own management* (informant 1, senior elected official).

These reflections showed an understanding and recognition of a need to collaborate with a wide range of actors to frame, investigate and handle complex challenges, such as NEET, in the community (informants 1 and 8). In addition, the senior elected official’s reflection about the traditional stakeholders in a policy development process can be understood as a critique of the classical bureaucratic logic that focuses on political and administrative actors. Instead, this argument revealed an ambition for building on the expertise of what were interpreted as relevant stakeholders, such as local businesses, labour market companies, citizens and the Norwegian Labour and Welfare Administration. Establishing the assignment committee can be interpreted as an expression of interactive leadership [25], where politicians exercise strategic leadership through stakeholder involvement and co-creation. Although the informants describe an engaged and positive atmosphere within the assignment committee, the interviews also reveal that there were diverse attitudes, perspectives and understandings of the problem among the 28 participants in the group (informants 2, 4, 7 and 8).

*I experienced a great deal of enthusiasm. But you know how it is, there were almost thirty people, each with their own backgrounds, viewpoints, and levels of enthusiasm. You have to avoid stifling any of that while trying to converge towards something that still creates a system, without anyone feeling like ‘I’m not being heard’. So, it was really a bit challenging* (informant 4).

This quote illustrates how the involvement of multiple actors challenges the problem framing, as different understandings and approaches are to be balanced to converge a common understanding of the problem and avoid derailment from initial problem framing and idea of solution.

### Raising and formulating NEET as a political challenge

We found the first official framing of NEET as a challenge for the local community in a budget proposal from 2018 that announced the suggestion of establishing an assignment committee to further investigate the problem (Document 1). The city council defined the mandate for the committee as ‘to come up with proposals for how the municipality can work better to include people in the workforce’ (Document 2, p. 3). This suggestion was politically formulated by the majority coalition and had not been processed by the administration (informant 5). In the proposal, NEET was formulated as a challenge that required new tools and improved systems:

*There are many good initiatives today, but we find that these initiatives and efforts are too fragmented. Therefore, we have allocated funding for an assignment committee that will assess how we can work smarter and more holistically to help more people realise their potential and assist more individuals in entering the workforce. […] The aim is for [Greenville] to be prepared with effective tools and improved systems to assist people in entering the workforce* (Document 1, Budget).

From the first formal initiative, NEET was framed as a structural problem for which the solution was connected to a broader system approach defined through ‘effective tools and improved systems’. The user perspectives and personal experiences with situations of NEET were also highlighted as rationale for raising NEET on the agenda:

*We were indeed concerned about young people’s experiences of being tossed around in the system. To address system changes [...], it was based not on theory but on real people struggling with real problems* (informant 1, senior elected official).

This specific framing of the problem increased engagement and involvement, highlighting that NEET situations were a system challenge. People who had experienced being in a NEET situation were invited by the consultancy to be part of the design-thinking process. Their stories and reflections made strong impressions and had a considerable impact on members of the assignment committee (informants 1, 4, 5, 6 and 7). In addition, demographic change in relation to an increasing elderly population actualized the demand for a larger workforce [49, 50]. Several national policy documents, reports and statistics focused on the challenges of demographic change and the consequences of a lack of competence and access to labour [8, 51, 52].

Along with national and regional statistics and analysis, the design-thinking process identified incentives and system structures as central components in addressing the NEET challenge (informants 1, 2, 4 and 6; Documents 3 and 4). The work of the assignment committee was formulated in a report recommending further follow-up over four themes: educational paths that lead to work, close follow-up of young people in NEET situations, a more flexible welfare and educational system, and organisational innovations to avoid dropout (Document 4). For further political follow-up, the report was revised into a political document by a senior executive, suggesting the establishment of a 5-year work inclusion program to deepen the work and implement the recommendations of the assignment committee. This suggestion was proposed by the administration and was unanimously approved by the city council (Document 8).

When investigating the agenda-setting process, we identified three crucial decisions that encompassed the problem and the solution. First, the city council decided to establish an assignment committee (Document 1), thereby acknowledging NEET as a public challenge. Second, the assignment committee report (Document 4) recommended further efforts. Third, there was a unanimous political decision by the city council to establish a 5-year work inclusion programme to deepen the work and implement the recommendations of the assignment committee (Document 8).

### Influential positions and agenda control

As presented, we have in this case identified two central policy entrepreneurs in the agenda-setting phase. First, the senior elected official was described and characterised as the central actor, initiator and architect (informants 1, 2, 5, 7 and 8) of raising NEET onto the governmental agenda and initiating the idea of system change as the solution. This senior elected official’s formal position as a political leader gave him legitimacy and therefore influence over the agenda (informant 5).

*It is part of the role of politicians or the mayor that you have a different kind of authority that is not necessarily related just to the fact that you are legally the political leader of a municipality but that you lead the city* (informant 1, senior elected official).

This statement reflects that formal positions and formal authority are not enough for being a political leader with agenda-setting power in a municipality. Senior elected officials also employ symbolic and reputational sources and need to develop soft powers and more transformational leadership, such as building a good reputation and relationships with networks and citizens and, thus, the target electorate. This constitutes an institutional feature of so-called embedded agency. Second, an external stakeholder was handpicked by the senior elected official to lead the committee on the basis of both his business experience and his independence from politics, political parties and the municipal administration:

*And I worked to get him to lead it, from the point of view that he had some good perspectives and also the ability to occasionally say the things that needed to be said, which people don’t dare to say* (informant 1, senior elected official).

The leader of the assignment committee gained influence and legitimacy through several of his former positions in local business. He had never been involved in local politics. Thereby, he could not be associated with any political agenda or conflict (informant 1). He was considered a respected business leader with extensive and relevant knowledge and expertise and was trusted by politicians from several parties on the city council (informants 1, 3 and 5). Choosing a non-politician and a consulting agency to manage the assignment committee’s work can be interpreted as a response to the embedded agency of the institutional features of the political system in local government, aiming to avoid political conflicts and achieve collaboration and consensus for the NEET challenge.

The two central policy entrepreneurs held positions with access and legitimacy to influence the political agenda (informants 2, 5 and 6). In addition to their influential positions, we found that strong agenda control was conducted by formulating a very precise mandate for the assignment committee and by inviting specific actors to participate.

*The background, the inspiration from Denmark, and what we had heard there were the basis for formulating the mandate for the Assignment committee. And it was quit precise mandate* (informant 1, senior elected official).

The detailed mandate from the city council to the assignment committee described the target group, how to organise the committee and a detailed committee budget (Document 2). This detailed mandate secured that the co-creation process of the assignment committee stayed in line with the original problem framing and idea of system change as the preferred solution. Some members of the assignment committee were critical of how specific statements and what they described as one-sided perspectives were emphasized in the co-creation process. They felt that conclusions were being drawn on a narrow or incomplete basis. Others described critical actors as resistant to change, and several informants noted tensions related to different understandings and approaches to the NEET topic (informants 2, 3, 4, 6 and 7). On the basis of the specifications in the mandate from the city council, the assignment committee leader took charge of the broader problem formulation process when discussions of perspectives and understandings unfolded (informants 4 and 7) and made decisive interventions.

The committee leader described the management role:

*You need the will to decide, you need decision-making authority, and you need decision-making ability; then you’ll come a long way* (informant 4, external stakeholder).

This statement describes features of leadership that were crucial when leading and manoeuvring the assignment committee consisting of multiple stakeholders with different perspectives.

### Surrounding turbulence

Parallel to the agenda-setting phase, the municipality was preparing a much-debated merger with two smaller neighbouring municipalities. This process took up a great deal of political and administrative focus and time during this period. It was described as difficult to involve the municipal organisation in the ongoing problem-framing process led by the assignment committee due to the reorganisation of the municipal administration:

*Throughout this period, it was extremely challenging to get the municipal organisation on board. […] Some [leaders] were on their way out, and some may have been on their way in. It was very challenging. So then, we conducted the process together with the consultancy – a process that was aimed much more at the partners and those who themselves had user experience* (informant 5, senior executive).

The difficulties and turbulence caused by the municipal merger process resulted in user experiences and external stakeholders’ perspectives playing a more significant role in framing and formulating the issue than they potentially would have if the municipal organization had not been preoccupied with the merger.

In addition to the municipal merger, there was a local election, where the outcome was that the central senior elected officials were not re-elected (Informant 5 and 8). A new political regime resulted in key policy entrepreneurs losing position and influence. A new political regime was instituted through cooperation between multiple minority fractions. The informants described a change in the political involvement and ownership of the idea due to the shift in the political regime (informants 1, 3, 6 and 8):

*It’s very unfortunate that it wasn’t the same [senior elected official] who handed over the project – or who received it, in a way – as the one who had initiated it. When you have such a change, you don’t know the project quite as well. It wasn’t you who set it in motion. And even if you’re enthusiastic and want to carry it out, there are these missing – blank gaps, perhaps – that prevent you from addressing things the way you usually would. […] So, I think, maybe, the learning point here is that when you initiate such things, there shouldn’t be an election coming* (informant 8, senior elected official).

The informants did not question the new political majority’s engagement with and efforts to improve the situations of young people experiencing NEET. Instead, they described a change in leadership and management of the policy development process when the central senior elected official left politics after the election (informants 1, 3, 6 and 8). This confirmed the senior elected official as an influential policy entrepreneur.

## Discussion

### The problem stream and definition of the policy problems

Shiffman [3] questions what influence the framing of health issues have on the likelihood of getting on the agenda. Béland and Katapally [7] argued that how a policy problem is defined is essential, as problem definitions that frame a collective problem rather than an individual one are more likely to reach the political agenda. In our study we found that the framing of NEET as a demographic and workforce challenge appeared crucial for getting local businesses involved in agenda setting and contributed to moving NEET from a low to a high politics issue. Framing NEET as a system challenge, rather than an individual problem, seemed to justify public intervention from a broad range of stakeholders [32]. Furthermore, understanding NEET as a system challenge functioned as a coalition magnet [33], whereby different stakeholders came to agree upon a common understanding of the challenge. Béland and Katapally [7] described how policy entrepreneurs strategically build coalitions and bring actors together to achieve support for a policy process. In our study, we found that the establishment of the assignment committee operationalised such strategic manoeuvres, initially through the formation of an assignment committee with multiple stakeholders from the community and then by hiring a consultancy to lead a design-thinking process to develop and anchor a common understanding of Greenville’s major challenge.

### The solution stream and development of a programme for system change

The intricate and multifaceted nature of public health issues, such as NEET, combined with limited scientific evidence on cause and effect, can lead to various interpretations that may challenge the initial policy idea of system change. Involving multiple stakeholders in policy formulation through the assignment committee’s work was in line with the new imperative in public health [23] and the smart city paradigm. Still, as described in the results, the broad involvement opened up a range of understandings and interpretations that potentially could have challenged the original policy idea, making it impossible to bring the idea onto the governmental agenda. In addition to the complex character of the public health challenge to be handled, we found that the high number of actors involved increased complexity and the need for centralised control in the agenda-setting phase. Keeping the agenda-setting process on track with the initial idea seemed crucial to avoid policy development derailment. It was paradoxical that a complex challenge was handled through the involvement of multiple actors, subsequently escalating complexity and entailing an increased need for agenda control.

Decision-making in the political arena involves a complex mix of economic, ideological and personal factors as well as evidence; turning a social problem into a political problem can be characterised by conflict [53]. When the policy formulation of a possible solution to handle situations of NEET was delegated to an assignment committee, the process was organised out of the political system, out of the local governmental arena and out of the municipal bureaucracy. Engaging an external stakeholder from outside politics and municipal administration to lead the work of the assignment committee can be understood as a strategy to reduce potential political and administrative tension and resistance. Outsourcing the formulation of the problem and solution away from the political agenda, ensured low political and administrative involvement and limited the potential for political turbulence and agenda-setting failure. Anchoring this process among important stakeholders through the design-thinking process increased the ambition of thinking outside the box and the innovativeness and boldness of the idea of a 5-year programme for developing a new urban ecosystem to improve the efforts aimed at young people in situations of NEET. It can also be interpreted as a strategy to cooperate in developing and maintaining a good relationship with relevant stakeholders in the community, and, therefore, it would be difficult for the political opposition to criticise the solution and the local ideas and understanding [15]. With the broad involvement of stakeholders in the assignment committee and the development of a coalition magnet and a common understanding of the problem and solution, young people in NEET situations, as a typical low politics issue [29], developed into a high politics issue [27], and it was easier to raise onto the governmental agenda. This can be understood as a strategy of interactive leadership [25], where politicians initiate co-creation, involving internal and external stakeholders, not only to identify new solutions but also to increase legitimacy for a new policy.

### The policy stream and the mood for system change

As described in the results, the policy stream was characterised by an international and national policy debate on concerns over projections of demographic and labour challenges. In addition, the challenge of young people in NEET situations, facing several European countries [54], strongly influenced the policy stream. Hence, there was a political mood for also formulating NEET as a policy problem at the local government level. In Nordic municipalities, the focus of the World Health Organization [55] on building resilient, healthy and smart cities started to accumulate over this period, and Greenville was a member of WHO’s healthy cities network, which focuses on how to develop service innovation and deal with challenges related to poor living conditions and social determinants of health. This international and national political mood was well suited for new policy ideas focusing on system challenges and the idea of approaching such problems with new and innovative ecosystem approaches.

Agenda setting in local governments unfolds in a complex context in which the surrounding turbulence, such as political shifts and unpredictable events, influences political processes [23]. As described in the results, a high level of turbulence and organisational noise dominated the political and administrative agendas. This seems to have constrained political and administrative involvement in the policy development process and enabled a window of opportunity for agenda setting through stakeholder involvement. Hence, there was limited interference and potential resistance from local politicians and municipal administration in the problem formulation process outsourced to the assignment committee. When the assignment committee report was raised as a political proposition on the political agenda, the problem and solution were anchored broadly among stakeholders outside the local government in such a way that it was difficult for the local politicians to oppose the idea from the targeted electorate.

### Policy entrepreneurs handling complexity

In this case study we found that complexity of NEET as a public health issue was handled by a clear problem definition connected to a potential solution. The challenge of young people in NEET situations was linked to the innovative solution by a Danish municipality, and coalition magnet [33] was established through multiple stakeholders’ involvement. The assignment committee and the consultant agency came to serve a dual strategy. Initiated by a senior elected official, functioning as the policy entrepreneur, the assignment committee became a formalized network arena for building the necessary coalition magnet involving multiple organizations, local businesses and volunteers. In addition, this made it possible for the policy entrepreneur to outsource a low politics issue from the governmental agenda to a network of multiple stakeholders to secure the necessary attention in the issue-framing and formulation phase of a typical low politics issue. It can be interpreted as a strategy from the senior elected official to secure control over the issue-framing and formulation phase concentrating on one specific issue, the NEET challenge, shielding it from party-political discussions and conflicts at an early stage to avoid the risk of lack of interest and willingness among politicians to deal with the issue. However, this approach may have implications for the following implementation of the policy. When political and administrative levels are excluded from the formulation phase, internal actors may have less ownership in the issue at stake, increasing the risk of later implementation failure.

Involving multiple stakeholders in the problem formulation escalated complexity further, as it entailed multiple perspectives and understandings. Hence, involving multiple stakeholders could threaten the understanding of problem and solution and the need for agenda control increased to maintain the coalition. The more pragmatic policy development at the local level, coupled with greater expectations for innovative policymaking and co-creation with a variety of internal and external actors, can result in multiple stakeholders potentially having agenda-setting power. This, in turn, may trigger the need for agenda control when specific issues are to be highlighted and prioritized. Our study indicates that one way to achieve agenda control was through a more selective inclusion and exclusion of actors involved in the agenda-setting phase. In addition, during the issue-framing phase, the matter was kept off the internal political agenda and organized outside the municipality, into a project group consisting of selectively included actors.

Given the institutional features of embedded agency, particularly the limited formal power and decision-making authority of political leaders in the local political system, agenda setting necessitates collaboration and collective decisions. Outsourcing problem formulation seems to avoid potential political conflicts, and involving multiple stakeholders in a co-creation process further developed a coalition magnet to legitimize the problem framing and defining the solution.

This article offers empirical insights into the role of policy entrepreneurs in addressing complex public health challenges at the local level. Our study illustrates that even within institutionalized settings, such as the local government setting, local politicians can strategically manoeuvre and navigate turbulent environments to bring complex public health challenges to the forefront of policy agendas.

Theoretically, the study contributes to agenda-setting theory by deepening our understanding of how agenda setting is related to the complexity of the issue at stake. In addition, the study illustrates how policy entrepreneurs in agenda setting are able to manoeuvre strategically even within highly institutionalized settings such as local governments.

Future studies on agenda setting in local government contexts should further examine the organisational and contextual conditions for agenda setting of complex public health challenges, considering the limits of this study. The findings in this study call for greater consideration of the embedded agency of agenda setting, underlining the limits of the merely strategic and agentic agenda setter. These findings should be further tested and developed comparatively across several cases.

Strengths and limitations

The strength of this case study lies in its capacity for in-depth analysis, which contributes to new insights and to the advancement of knowledge of local government agenda setting and decision-making [36]. However, the findings from this case study are challenging to generalize owing to the unique context and relationships inherent to the chosen case. Our findings are highly context-dependent, limiting the generalizability of results [56, 57]. Still, the in-depth insights from this study contribute new knowledge about agenda setting of complex challenges in local government contexts.

This case study has some obvious limitations, as temporal constraints on observations and data collection limited sampling. In addition, the number of informants, documents, and observations included were limited. These selectivity issues impacted the data and results. 

The interviews were conducted retrospectively and gave rich data on the agenda-setting process and timeline. However, retrospective interviews involve a risk of selective memories influenced by later happenings. Hence, the manoeuvres in our case may appear more strategic and planned than reality. Nevertheless, the retrospective interviews gave deep insights into the agenda-setting process, which was also traced from documents. Triangulating data from documents and interviews increased the validity of our study [56, 58].

## Conclusion

This paper adds to literature on public health policymaking and especially the agenda setting of complex public health issues. In this study of agenda setting and the role of policy entrepreneurs we identified how complexity was handled to raise low politics issue, young people in NEET situations, onto a local governmental agenda. Our findings indicate that agenda setting in local governments involves translating complex public health challenges into feasible solutions, demonstrating the policy entrepreneurs’ ability to transform complexity into manageable narratives. Policy entrepreneurs’ ability to create and maintain coalition magnets through broad involvement of stakeholders was crucial to build a legitimacy base and get the public health challenge of NEET on the governmental agenda. The broad involvement was not only crucial for building support and legitimacy for proposed solutions but also increased the need for agenda control to maintain problem framing and coalition agreement.

Complex public health challenges call for policy entrepreneurs with influential positions and legitimacy who are able to strategically manoeuvre to gain agenda control through the systems of multiple actors and turbulent surroundings. Understanding the embedded agency in agenda setting is essential, recognizing the limitations of purely strategic approaches and considering the broader context and dynamics at play. For future studies, applying complexity theory may offer a valuable perspective for analysing how agenda setting unfolds in the complex and turbulent context of local governments. Further research on comparative cases, including cases outside the Nordic context, as well as applying a process-tracing design could contribute important in-depth insights to our findings related to agenda control, temporality and complexity in governmental agenda setting.

## Supplementary Information

Supplementary Material 1. Supplementary file 1: Table of Documents. Table S1, provides a table of the documents applied for and referenced to in this study. The documents are numbered, dated, named and briefly summarized.

Supplementary Material 2. Supplementary file 2: Analyses. Table S2, provides a table of themes and codes constructed in the thematic analysis.

## Data Availability

No datasets were generated or analysed during the current study.
