# Supplementary material for: Raising complex public health challenges on local government agendas: a Norwegian case study
Source: Health Res Policy Syst. 2025 Jun 18;23:79. doi: 10.1186/s12961-025-01347-3 (PMC12177986; doi:10.1186/s12961-025-01347-3)
Supplement: Supplementary file 1 — Supplementary Material 1. Supplementary file 1: Table of Documents. Table S1, provides a table of the documents applied for and referenced to in this study. The documents are numbered, dated, named and briefly summarized. [file 12961_2025_1347_MOESM1_ESM.docx]

## Supplementary file 1: Table of documents

**Table S1: Table of documents**

| **Document number** | **Year** | **Document**  **City council** | **Description** |
| --- | --- | --- | --- |
| 1 | Dec. 2018 | Budget  Proposition | The idea of an assignment committee is launched by city council position in their budget proposition. |
| 2 | May 2019 | Mandate | City council approve a mandate for the assignment committee. |
| 3 | April 2020 | Presentation by Consultant Agency | Power Point presentation of insights gained through Design thinking process of the assignment committee. |
| 4 | May 2020 | Assignment committee Report | The work process and recommendations from the assignment committee is presented in a report, handed over to the administration and introduced to the city council. |
| 5 | Aug. 2020 | Suggested follow-up of report | Follow-up suggestion from the administration. |
| 6 | Oct. 2020 | City Council debate | Summary of debate in city council. 40 verbal remarks addressing follow up of assignment committee recommendations. |
| 7 | Dec. 2020 | Summary 40 verbal remarks | Summary of 40 verbal remarks from city council debate of assignment committee report and recommendations. Remarks are grouped and sorted. |
| 8 | April 2021 | Establishing 5-year Work Inclusion Programme | Based on the assignment committee report, administration suggests to city council to establish a 5-year programme aiming to increase work inclusion of young NEETS. |
| 9 | May 2021 | Comparing Danish municipality and ‘Greenville’ | Note comparing the different framework for ‘Greenville’ and the Danish municipality, a review of research related to the effects of the Danish model and a recommendation of what ‘Greenville’ could learn from the Danish municipality. |
