# Supplementary material for: Raising complex public health challenges on local government agendas: a Norwegian case study
Source: Health Res Policy Syst. 2025 Jun 18;23:79. doi: 10.1186/s12961-025-01347-3 (PMC12177986; doi:10.1186/s12961-025-01347-3)
Supplement: Supplementary file 2 — Supplementary Material 2. Supplementary file 2: Analyses. Table S2, provides a table of themes and codes constructed in the thematic analysis. [file 12961_2025_1347_MOESM2_ESM.docx]

Supplementary file 2: Analyses

|  | **Themes** | **Codes** |
| --- | --- | --- |
| ***Predefined Themes*** | ***Problem stream*** | Problem framing |
|  | ***Solution stream*** | Background |
|  |  | Inspiration |
|  | ***Policy stream*** | Ambitions, local and national |
|  |  | Resistance |
|  |  | Window of Opportunity |
|  | ***Policy entrepreneur*** | Agenda control |
|  |  | Influence |
|  |  | Leadership |
|  |  | Performance |
| **Constructed Themes** | **Assignment Commitee** | Invited members |
|  |  | Mandate |
|  | **Municipality 3.0** | Complexity |
|  |  | Consultant agency as process driver |
|  |  | Design Thinking |
|  |  | User involvement |
|  | **Politics** | Interaction Politicians and Administration |
|  |  | Political Engagement |
|  |  | Political Follow-up |
|  |  | Political Steering |
|  | **Surrounding Turbulence** | Legal Framework |
|  |  | Local Election |
|  |  | Municipal Merge |
|  | **Implementation** | Administration ownership |
|  |  | Programme as Solution |
|  |  | Time lap |
